# miRNAs Predicted to Regulate Host Anti-viral Gene Pathways in IPNV-Challenged Atlantic Salmon Fry Are Affected by Viral Load, and Associated With the Major IPN Resistance QTL Genotypes in Late Infection

**DOI:** 10.3389/fimmu.2020.02113

**Published:** 2020-09-11

**Authors:** Nardos Tesfaye Woldemariam, Oleg Agafonov, Hilde Sindre, Bjørn Høyheim, Ross D. Houston, Diego Robledo, James E. Bron, Rune Andreassen

**Affiliations:** ^1^Department of Life Sciences and Health, Faculty of Health Sciences, OsloMet – Oslo Metropolitan University, Oslo, Norway; ^2^Department of Core Facilities, Bioinformatics Core Facility, Institute of Cancer Research, Radium Hospital, Oslo University Hospital, Oslo, Norway; ^3^Department of Fish Health, Norwegian Veterinary Institute, Oslo, Norway; ^4^Department of Basic Sciences and Aquatic Medicine, Faculty of Veterinary Medicine, Norwegian University of Life Sciences, Oslo, Norway; ^5^Division of Genetics and Genomics, The Roslin Institute and Royal (Dick) School of Veterinary Studies, The University of Edinburgh, Edinburgh, United Kingdom; ^6^Faculty of Natural Sciences, Institute of Aquaculture, University of Stirling, Stirling, United Kingdom

**Keywords:** Atlantic salmon, microRNA, IPNV, immune response, host-virus interactions

## Abstract

Infectious pancreatic necrosis virus (IPNV) infection has been a major problem in salmonid aquaculture. Marker-assisted selection of individuals with resistant genotype at the major IPN quantitative trait locus (IPN-QTL) has significantly reduced mortality in recent years. We have identified host miRNAs that respond to IPNV challenge in salmon fry that were either homozygous resistant (RR) or homozygous susceptible (SS) for the IPN-QTL. Small RNA-sequenced control samples were compared to samples collected at 1, 7, and 20 days post challenge (dpc). This revealed 72 differentially expressed miRNAs (DE miRNAs). Viral load (VL) was lower in RR vs. SS individuals at 7 and 20 dpc. However, analysis of miRNA expression changes revealed no differences between RR vs. SS individuals in controls, at 1 or 7 dpc, while 38 “high viral load responding” miRNAs (HVL-DE miRNAs) were identified at 20 dpc. Most of the HVL-DE miRNAs showed changes that were more pronounced in the high VL SS group than in the low VL RR group when compared to the controls. The absence of differences between QTL groups in controls, 1 and 7 dpc indicates that the QTL genotype does not affect miRNA expression in healthy fish or their first response to viral infections. The miRNA differences at 20 dpc were associated with the QTL genotype and could, possibly, contribute to differences in resistance/susceptibility at the later stage of infection. *In silico* target gene predictions revealed that 180 immune genes were putative targets, and enrichment analysis indicated that the miRNAs may regulate several major immune system pathways. Among the targets of HVL-DE miRNAs were IRF3, STAT4, NFKB2, MYD88, and IKKA. Interestingly, TNF-alpha paralogs were targeted by different DE miRNAs. Most DE miRNAs were from conserved miRNA families that respond to viral infections in teleost (e.g., miR-21, miR-146, miR-181, miR-192, miR-221, miR-462, miR-731, and miR-8159), while eight were species specific. The miRNAs showed dynamic temporal changes implying they would affect their target genes differently throughout disease progression. This shows that miRNAs are sensitive to VL and disease progression, and may act as fine-tuners of both immediate immune response activation and the later inflammatory processes.

## Introduction

MicroRNAs (miRNAs) are small (20–24 nts) non-coding RNAs that regulate gene expression at the post-transcriptional level by binding to mRNA transcripts (target genes) in a sequence specific manner (1, 2). As part of the miRISC complex, they fine-tune gene expression by degrading target mRNAs or by interfering with their translation (3–5). There are several hundred miRNA genes in vertebrate species (http://mirbase.org/), and each of these contribute to the regulation of multiple biological processes (6–8). Several studies in higher vertebrates indicate that the host immune defense against viral infections are among the processes regulated by miRNAs (9–11).

Our previous study on miRNAs and host-virus interactions in Atlantic salmon revealed several evolutionary conserved miRNA families that responded to salmonid alphavirus (SAV) infection (12). Among these conserved miRNAs were miR-21, miR-146, miR-181, and the clustered miRNAs miR-462 and miR-731. Some of these also revealed differences in miRNA expression when fish were challenged by SAV-subtypes with diverging mortality (12). Although the functional analysis of these miRNAs is still in progress, the responding miRNAs were predicted to target several genes involved in innate immune and inflammatory responses, suggesting that these host miRNAs were directly involved in regulation of the Atlantic salmon antiviral immune responses. Andreassen and Høyheim (13) proposed a model which suggests a role for miRNAs as fine-tuning modulators of immune responses during viral infections in teleost. The model suggests that at normal healthy state, the constitutive expression of miRNAs targeting immune activator transcripts restricts the triggering of an immune response. When a viral infection occurs, miRNAs can take on a dual role that help to fine-tune the immune response in order to benefit the host. Downregulation of host miRNAs that target immune response activator genes at the early stage of viral infection may, for example, lead to an increased expression of the immune response activators, and consequently promote activation of the host immune responses. Upregulated expression of miRNAs that target activators of inflammation in the later stages of infection could, on the other hand, balance the magnitude of the response, and help prevent escalation of inflammatory processes to a level that could be harmful to the host (13).

Infectious pancreatic necrosis virus (IPNV), a member of the virus family Birnaviridae, is a non-enveloped birnavirus causing infectious pancreatic necrosis (IPN). IPN is a disease associated with high mortality mainly in juvenile salmonids, including Atlantic salmon fry (14), but considerable mortalities from IPN have also been reported in Atlantic salmon post-smolts (15). In recent years, the number of outbreaks and losses due to IPN have been significantly reduced (16). This decline can mainly be attributed to the discovery of a major quantitative trait locus (IPN-QTL) associated with a heritable difference in IPN-resistance (17, 18), that has been applied in marker-assisted breeding of IPN resistant fish. A single-nucleotide polymorphism (SNP) found in the epithelial cadherin gene (*Cdh1*) has been suggested as contributing to the resistance associated with the IPN-QTL (19). The *Cdh1* gene encodes a calcium-dependent cell adhesion protein with key roles in epithelial cell behavior, tissue formation, and suppression of cancer (20). A significant association between the IPN-QTL and the SNP has been reported (19). Results from an *in vitro* study applying an immunofluorescence detection of IPNV and the *Cdh1-1* protein bound to IPNV suggested that entry of the virus was facilitated in hepatocytes from individuals with the susceptible variant of the SNP, while the ability of the virus to enter hepatocytes was restricted in individuals with the resistant SNP variant (19). The findings suggested that the functional role of the gene involves internalization of IPNV virions into the host (19). However, subsequent research has shown that virions can enter the cells of resistant salmon (21), and that the clathrin-mediated endocytosis proposed in (19) is not the entry mechanism used by IPNV to enter Atlantic salmon cells in culture (22). Therefore, the nature of the mechanisms leading to the heritable differences in resistance to IPN associated with the IPN-QTL are not fully understood (19, 21).

IPNV is one of the most intensely studied viruses of fish, and some studies have investigated the gene expression responses to IPNV (23–29). Recently, Robledo et al. (21) studied the gene expression profiles of IPNV challenged Atlantic salmon fry from families showing large differences in susceptibility to IPN. Their findings demonstrated significant differences in the immune responses between families that were classified as either “IPN-resistant” or “IPN-susceptible.” The susceptible families were characterized by a large early innate immune response, while a moderate, putatively macrophage-mediated inflammatory response was characteristic for the IPN-resistant families. The information from these transcriptomic studies has provided valuable insights into some of the mechanisms of host immune responses to IPNV. In spite of the emerging evidence that miRNAs are important regulators of host immune responses, no studies have focused on the roles of miRNAs in host response to IPNV and whether they contribute to susceptibility or resistance to disease.

The aim of this study was to identify Atlantic salmon miRNAs that are differentially expressed in response to IPNV challenge (DE miRNAs). Samples were analyzed at three time points post challenge to identify miRNAs responding at different infection stages following the viral challenge. In contrast to the SAV study where we investigated the miRNA response in fish infected by different virus subtypes (12), this study aimed at investigating whether there was any association between miRNA response and viral copy number, or if there were differences in response associated with the IPN-QTL. Materials were sourced from a family where the selected offspring were homozygous for either the resistant or the susceptible genotype at the IPN-QTL. Small RNA sequencing followed by miRNA expression analysis identified miRNAs that showed modulation in their expression associated with viral load and/or genotype at the IPN-QTL. Finally, the regulation of host antiviral immunity against IPNV by DE miRNAs was further explored by *in silico* target gene prediction to identify miRNA-target gene interactions associated with immune response, inflammation and/or apoptosis.

## Materials and Methods

### Materials

The salmon fry were collected as part of a larger challenge experiment described in Houston et al. (30). The challenges were performed at the Center for Environment, Fisheries and Aquaculture Science (Cefas) under the approval of their ethical review committee and complied with the Animals Scientific Procedures Act. The fish were sampled and euthanized using a procedure specifically listed on the appropriate Home Office (UK) license.

The Atlantic salmon fry were from family C described in Houston et al. (30). Parents were heterozygous (RS) at the IPN-QTL (17). The offspring included in this study were of either the homozygous susceptible (SS) or homozygous resistant (RR) genotype at the IPN-QTL. The QTL-genotyping was carried out as described in Houston et al. (30). Fry were bath challenged with IPNV isolate V0512-1 (serotype A2 (Sp)). They were sampled at 1 day post challenge (1 dpc), 7 days post challenge (7 dpc) and 20 days post-challenge (20 dpc). Twelve fry, six with the homozygous resistant genotype (RR) and six with the homozygous susceptible genotype (SS), were selected from each of the time points post-challenge (*n* = 36). Healthy controls were sampled prior to bath challenge (*n* = 11). The total materials were, thus, 47 samples including the healthy controls. Houston et al. (30) reported the mortalities from the challenge trials. Mortalities were on average 63% if individuals were SS (homozygous susceptible genotype), while it was 5% if individuals were RS (heterozygous genotype) and 0% if fish were RR (homozygous resistant genotype). None of the control fish developed IPN or died (mortality = 0%). Detailed descriptions of the rearing conditions of the fry, virus preparation and challenge protocol are given in Houston et al. (30).

### Methods

#### Small RNA Isolation

Total RNA was isolated from the whole fry samples using TRI reagent (Sigma–Aldrich®, St. Louis, MO, USA) following the manufacturer's instructions as described in Houston et al. (30). The whole fry homogenate was used in the total RNA extraction. The RNA quality and quantity were determined using spectrophotometry (NanoDrop ND-1000, Thermo Scientific, Wilmington, USA) and agarose gel electrophoresis. The A260/280 ratios were higher than 1.9 in all the samples (see Table S1).

#### Quantitation of Viral Loads by RT-qPCR

Viral loads of IPNV were determined by quantitative RT-qPCR using QIAGEN® oneStep RT-PCR kit (Qiagen GmBH, Hilden, Germany). The primers and probes were designed to amplify a fragment of 109 bp in the VP3 region of IPNV serotype Sp, and samples were analyzed as described in Orpetveit et al. (31). Samples with quantification cycle (Cq) values of ≤ 40 were considered positive. Mann-Whitney U tests performed by SPSS version 24.0 (SPSS Inc., Chicago, IL, USA) were used for statistical comparisons of the viral loads (determined through Cq values) between the IPN-QTL resistant (RR) and IPN-QTL susceptible (SS) groups. *P* < 0.05 were considered significant.

Confirmation of the IPNV type used for the challenge was obtained by sequencing the capsid protein VP2 using primers described in Santi et al. (32). The sequencing was performed using the BigDye™ Terminator v3.1 Cycle Sequencing Kit and a 24-capillary 3500xL Genetic Analyzer, both from Applied Biosystems (Foster City, CA, USA) as described in (32).

#### Library Construction, High-Throughput Sequencing, and DESeq2 Analysis

Small RNA libraries were constructed and sequenced at the Norwegian High-Throughput Sequencing Center (NSC). The NEBNext® Multiplex Small RNA Library Prep Set for Illumina (New England Biolabs, Inc. Ipswich, MA, USA) was used in the preparation of 47 libraries from the 36 IPNV challenged samples and the 11 controls. Total RNA, 1 μg per sample, was used as input in the library preparation in accordance with the manufacturer's protocols. Small RNAs isolated from the 47 whole fry were ligated with 3′ and 5′ RNA adapters, followed by reverse transcription and PCR enrichment using barcoded RT-primers. The cDNA products were purified using 6% polyacrylamide gels, and size selection of fragments (~145–160 bp) was carried out to enrich for miRNAs. The sequencing was performed at NSC on a NextSeq 500 from Illumina, producing 75 base single-end reads.

Data processing of the raw reads followed the procedures described in Woldemariam et al. (33). The raw reads were quality checked using FASTQC (v.0.11.55) (34) and processed with cutadapt (v.1.18) (35). This process removed low quality reads, adapter-only sequences and reads outside the expected size range of mature miRNAs (18–25 nts). Subsequently, the clean sequence reads (18–25 nts) were mapped to a genome index consisting of all known mature miRNAs in Atlantic salmon (33) using STAR (v.2.5.2b) (36). All sequencing reads have been submitted to the National Center for Biotechnology Information (NCBI), to the public repository for the next-generation sequence data, the Sequence Read Archive (SRA) (https://www.ncbi.nlm.nih.gov/sra/).

The alignment files were further processed in R using the *featureCounts* function from the Rsubread package (37) to produce count matrices. These count tables were used as input in the Bioconductor package *DESeq2* (v.1.20.0) in R (38) to analyze differential miRNA expression. Differentially expressed miRNAs (DE miRNAs) at each of the time points (1, 7, and 20 days post challenge) were identified by comparing control group and all challenged individuals at each time point. Furthermore, IPN-QTL resistant (RR) and IPN-QTL susceptible (SS) groups were also compared at each time point to identify miRNAs associated with IPN-QTL genotype. Differentially expressed miRNAs were defined as all miRNAs with Benjamini-Hochberg adjusted *p* ≤ 0.05, log2 fold change threshold value of at least ≤ −1.0 or ≥ 1.0, and with normalized read counts (from *DESeq2* analysis) ≥ 5 in each sample. Unsupervised hierarchical clustering analysis was then performed using the *hclust* function from the *stats* package in R, with the Euclidean distance metric and Ward's method for clustering (39). Heatmaps and cluster dendograms with the differentially expressed miRNAs grouped by the clustering analysis were plotted using the *heatmap.2* function in the R package *gplots* (v.3.0.1.1) (40).

#### Target Gene Predictions

Target gene prediction (*in silico* analysis) was carried out with RNAhybrid (41). The mature sequences from the DE miRNAs [mature sequences given in Woldemariam et al. (33)] were tested against 3'UTRs from all Atlantic salmon mRNA transcripts in the NCBI Reference Sequence database (Refseq) (42). The following parameters were applied in the RNA hybrid analysis: helix constraint 2–8, no G: U in seed and minimum free energy threshold−18 kcal/mol. Gene functions of predicted target genes were retrieved from the Universal Protein Resource (UniProt) database https://www.uniprot.org/ (43). Based on the GO annotations, the subsets of target genes relevant to immune response were identified. Cross-reference links for these genes in Uniprot were used to retrieve organism-specific gene pathways from the online resource Kyoto Encyclopedia of Genes and Genomes (KEGG) pathway database (https://www.genome.jp/kegg/pathway.html) (44). These genes were also used as input in gene pathway enrichment analyses applying the Enrichr tool (http://amp.pharm.mssm.edu/Enrichr/). Results were then filtered by organism to rank gene pathways present in Atlantic salmon by their adjusted P-values (Q-values) and combined score.

#### Genes With Expression Changes at 20 dpc

Dr. Diego Robledo and colleagues provided information from a microarray experiment identifying genes responding to IPNV challenge at 20 dpc (log fold change of gene expression level at least ≤ −1.0 or ≥ 1.0). The fry used in this microarray experiment were the same materials as used in the current study (unpublished data by Robledo et al.). A subset (*n* = 25) of the genes identified as IPNV responsive at 20 dpc were among those immune genes that were predicted as target genes of the miRNAs identified in family C from the comparison of IPN-QTL susceptible (SS) and IPN-QTL resistant (RR) individuals at 20 dpc. These 25 immune genes and the miRNAs all showed expression changes following IPNV challenge in same materials and at the same time point. We therefore compared the magnitude and direction of the expression changes in these genes and miRNAs to gain further insights into their putative interactions.

## Results

### Measurements of Viral Loads

IPNV RNA was successfully detected and quantified by RT-qPCR. Six of the 11 controls showed very low (Cq > 37), but still positive results of viral presence. There were no differences in miRNA expression between these six controls (Cq > 37) and the other negative controls (Cq > 40) (data not shown) indicating that these controls had not been exposed to IPNV at the initial stage of the challenge. Thus, all 11 samples were used as controls in the miRNA expression analyses (see Differentially expressed miRNAs (DE-miRNAs) show dynamic expression patterns post challenge and miRNAs associated with IPN-QTL genotype and prolonged high viral loads), five of which were homozygous resistant (RR) and six that were homozygous susceptible (SS) for the IPN-QTL. The presence of very low levels of IPNV could be caused by contamination of some controls somewhere in the analysis pipeline. To elucidate whether this could be the cause, the viral capsid protein VP2 sequence was amplified and sequenced in the positive controls and in some samples from challenged fish with high viral loads collected at later time points. The VP2 sequences from all these samples were identical supporting a contamination of these controls with virus from the same source as was used in the challenge trial.

**Figure 1:**
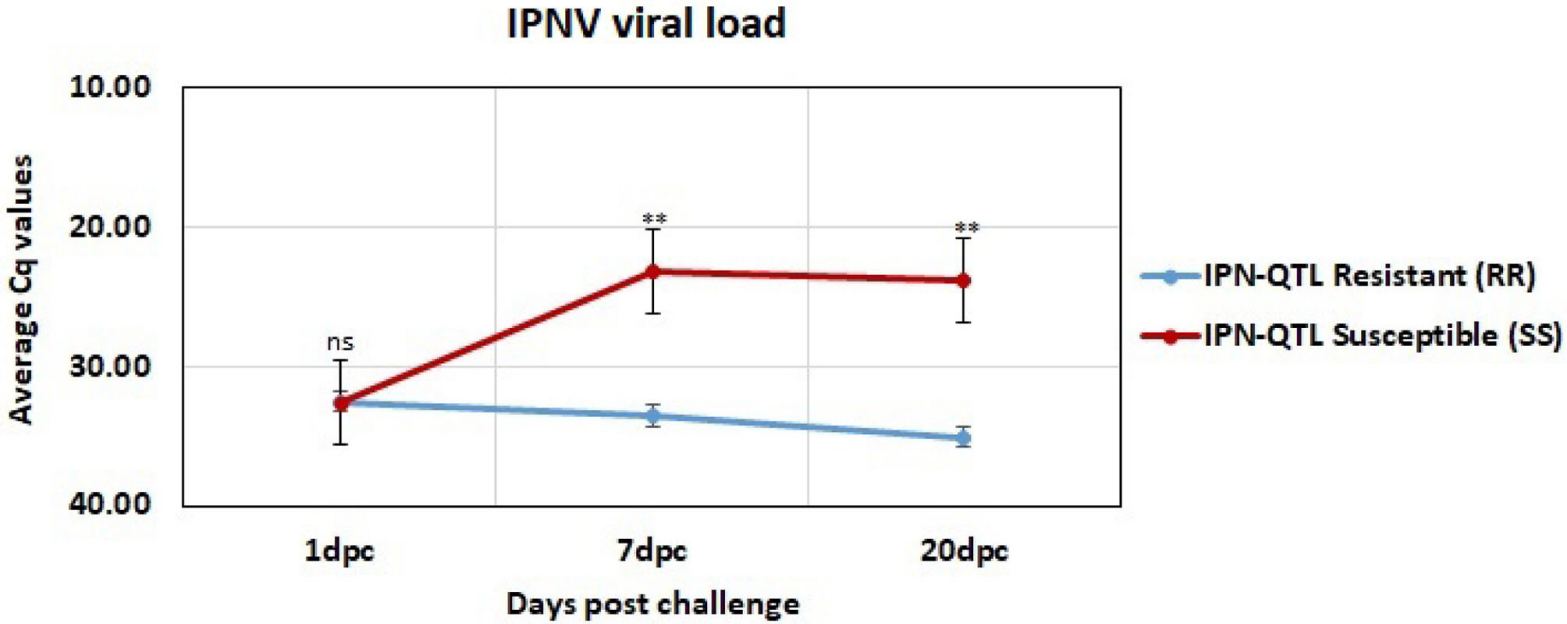
Average viral load in IPN-QTL resistant (RR) and IPN-QTL susceptible (SS) fry at 1, 7, and 20 dpc. The significance symbols represent the p-values for Mann-Whitney *U*-test between the RR and SS (ns: means *p* > 0.05 (not significant), **: means *p* ≤ 0.01).

The viral load was measured in all challenged individuals at 1, 7, and 20 dpc. These measurements showed that IPNV could be detected in both RR and SS fish as early as 1 dpc (Figure 1). The 1 dpc measurements were similar in all samples with an average Cq value of 32.5 in both RR and SS. Thus, the response to challenge measured by their viral load did not differ between IPN-QTL genotypes at this early stage of infection. There were, however, significant differences between the QTL groups (RR and SS) at both 7 and 20 dpc, with an ~1,000-fold higher viral load in the IPN-QTL SS group than in the IPN-QTL RR group (*p* ≤ 0.01). The higher viral loads observed in the SS group was in accordance with the expectations that individuals that were homozygous for the susceptible QTL-genotype would develop a more severe viral infection.

### RNA Library Preparation and Small RNA Sequencing

To identify Atlantic salmon miRNAs responding to IPNV infection RNA was successfully extracted from 47 whole fry and all samples were subsequently small-RNA sequenced. A total of 799,382,140 raw reads were obtained from the IPNV challenged samples (*n* = 36) and the healthy controls (*n* = 11). The quality filtered (Phred score > 32), adapter and size trimmed reads for each sample ranged from 2.9 to 15.3 million. The proportion of clean reads that mapped uniquely to the reference Atlantic salmon miRNAome ranged from 56.3–77.8%. An overview of sample origin, RNA concentration, read numbers, reads mapped to miRNAs and the SRA accession numbers are given in Table S1. Overall, the sequence reads produced from each sample were of high quality and suitable for differential expression analysis.

### Differentially Expressed miRNAs (DE-miRNAs) Show Dynamic Expression Patterns Post Challenge

To identify miRNAs that showed expression changes in response to IPNV infection, we initially compared the expression of miRNAs in controls against samples from 1, 7 and 20 days post challenge. This analysis revealed 72 mature miRNAs belonging to 42 miRNA families that were differentially expressed (DE) relative to controls on at least one of the three time points. Additionally, to identify miRNAs that were associated with resistance/susceptibility, we also compared the IPN-QTL susceptible genotype groups (SS) against IPN-QTL resistant genotype groups (RR) at all timepoints. This was done to reveal miRNAs that could putatively be involved in mechanisms leading to resistance in the RR group and susceptibility in the SS group. Differences in miRNA expression between RR and SS were only found at 20 dpc. There were 38 such miRNAs, but 28 of these miRNAs were also among the 72 miRNAs identified in the initial analysis of different time points against controls. An overview of all the 82 DE genes identified in the time point analysis' and comparisons between genotypes are given in Table S2. The supplementary table show results from the SS groups and the RR groups separately. A detailed description of the 38 miRNAs associated with differences in resistance/susceptibility (QTL-genotypes) is given in section miRNAs associated with IPN-QTL genotype and prolonged high viral loads.

**Figure 2:**
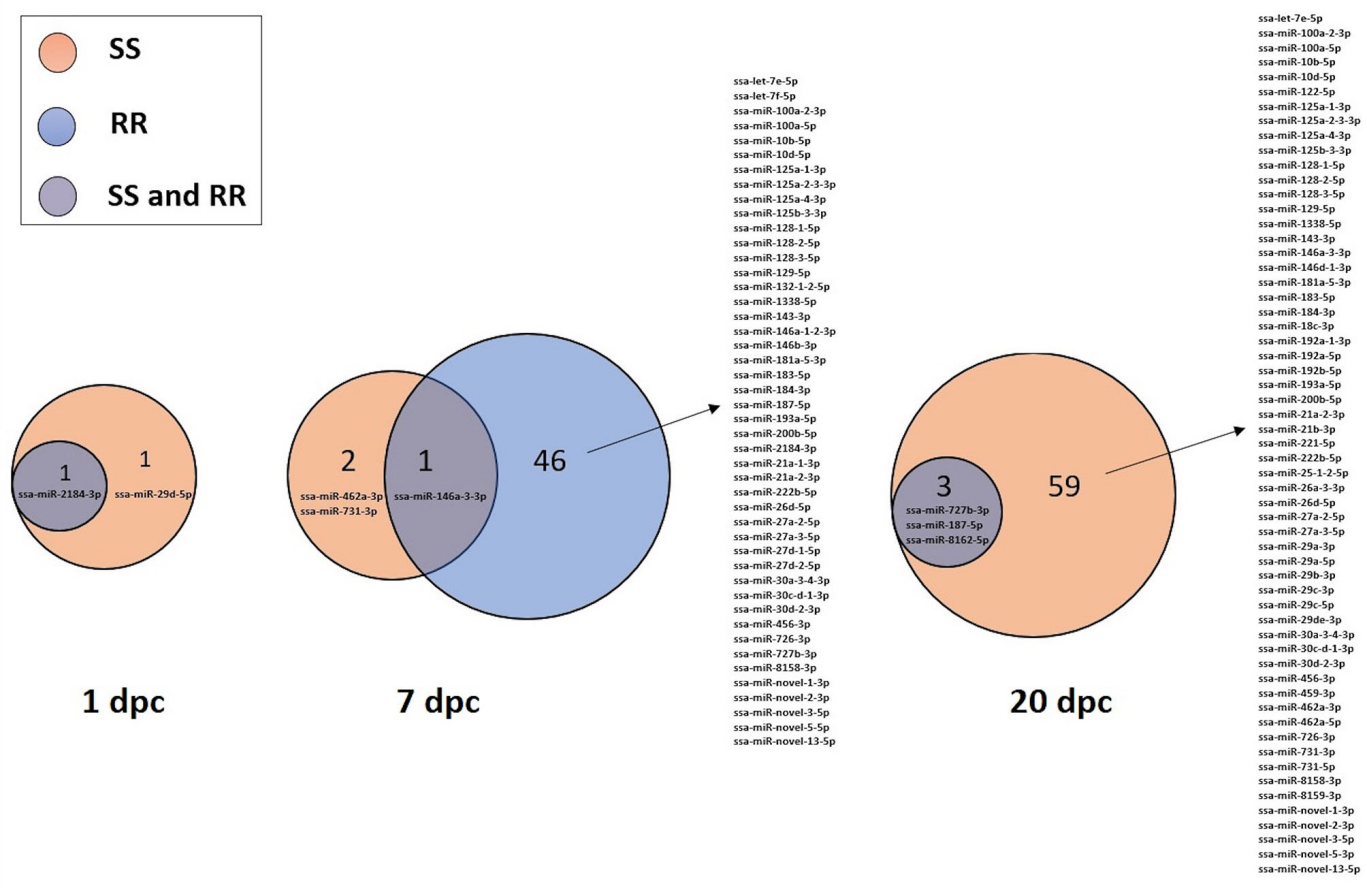
Venn diagrams showing the number of miRNAs belonging to either the SS and/or the RR group that were significantly different to controls at either 1, 7, or 20 days post IPNV-challenge (dpc).

**Figure 3:**
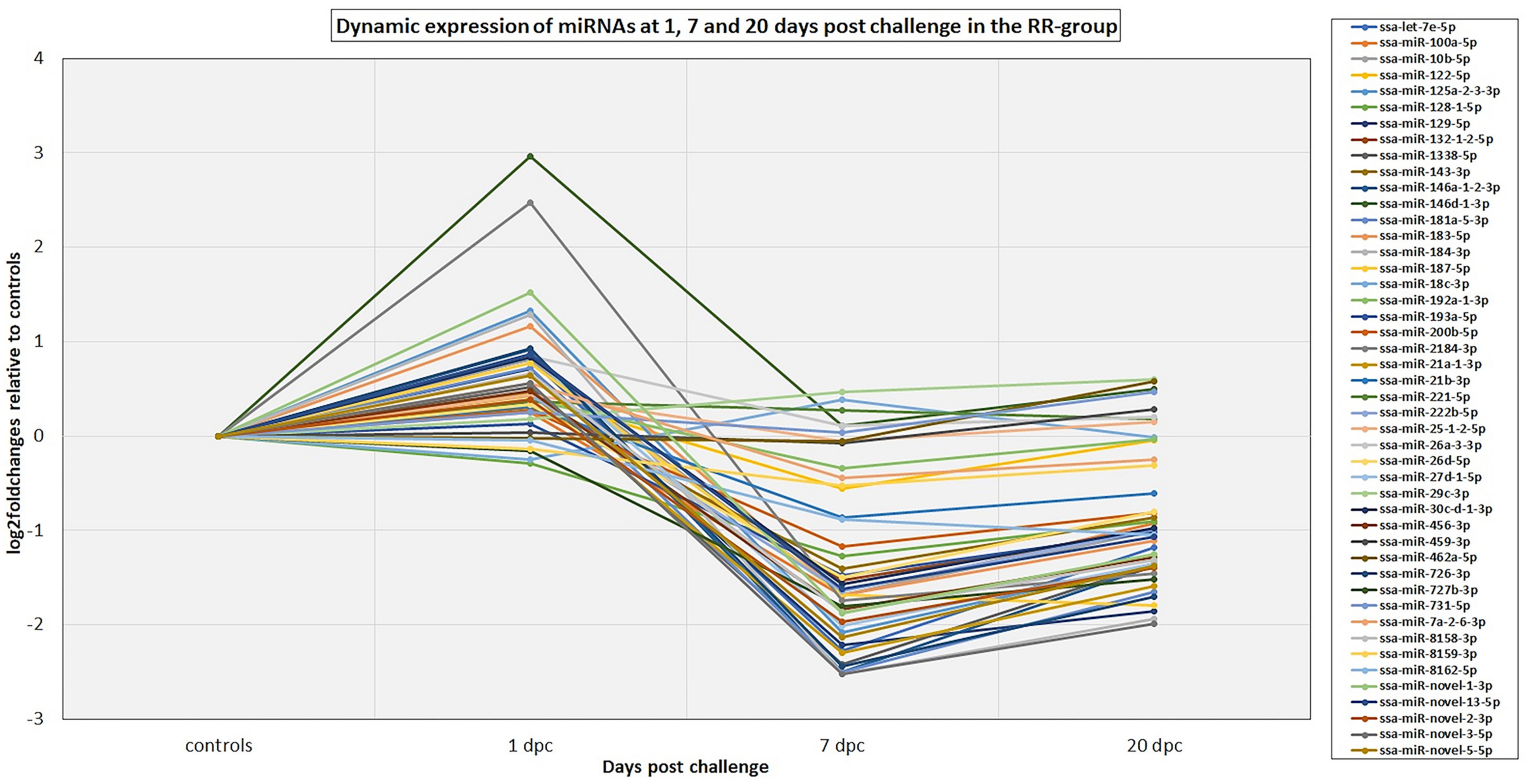
The dynamic expression profiles of miRNAs differentially expressed at either 1, 7, and/or 20 dpc in the RR group. The plot shows the expression changes (log2foldchanges) for each of the miRNAs at each time point post IPNV-challenge relative to controls. Several of the miRNA genes in the same miRNA family showed similar dynamics and to simplify the plot only the major expressed mature member of each family is presented in the plot. The complete results are given in Table S2.

**Figure 4:**
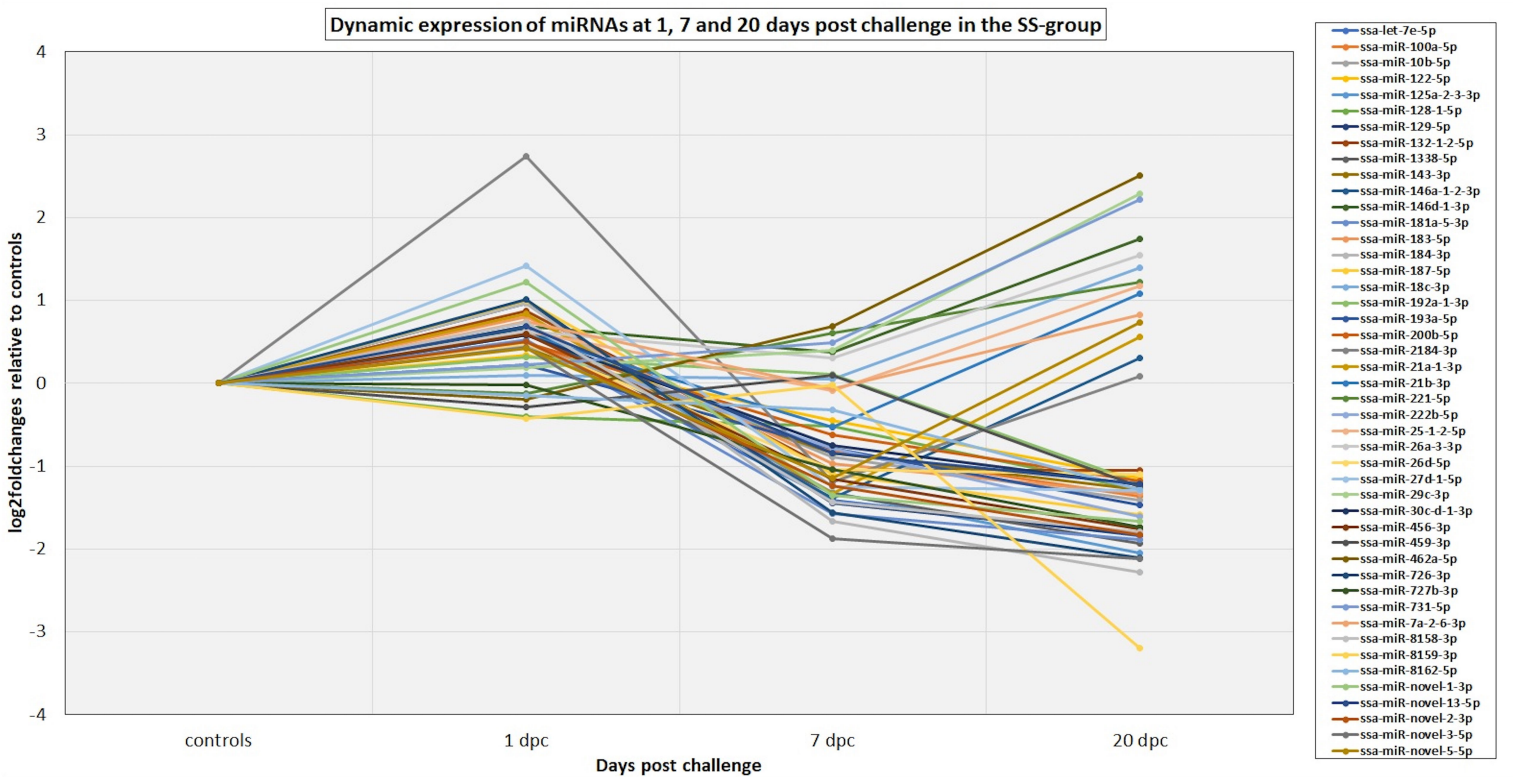
The dynamic expression profiles of miRNAs differentially expressed at either 1, 7, and/or 20 dpc in the SS group. The plot shows the expression changes (log2foldchanges) for each of the miRNAs at each time point post IPNV-challenge relative to controls. Several of the miRNA genes in the same miRNA family showed similar dynamics and to simplify the plot only the major expressed mature member of each family is presented in the plot. The complete results are given in Table S2.

The 82 DE miRNAs showed dynamic expression patterns compared to controls. The RR group changes were slightly more pronounced at 7 dpc, and more miRNAs were significantly changed according to our thresholds (see methods) in this group than in the SS group. The changes of the SS group miRNAs were, however, very similar to the RR group. Although they were not significantly different to controls they changed in same direction (less expression than in controls) as in the RR group and were not significantly different to this group. On the other hand, the SS group showed the largest changes compared to controls at 20 dpc, and, in many cases, were also significantly different to the RR group. An overview of number of mature miRNAs different to controls for each of the QTL groups at each time point is given in Figure 2. In addition, the dynamic expression patterns for each of the QTL groups are illustrated in Figures 3, 4. As shown in Figure 3, the 47 DE miRNAs in the RR group at 7 dpc (Figure 2) were down-regulated while the SS group revealed very similar, although slightly less pronounced, changes in same direction as in the RR group (7 dpc, Figure 4). One miRNA, ssa-miR-2184-3p, showed more than a 4-fold increase in expression in both the RR and SS group at 1 dpc, while ssa-miR-29d-5p, that also showed increased expression in both groups at 1 dpc, was only significantly different to controls in the SS group (Figures 2, 4). There were 62 mature miRNAs differently expressed compared to controls at 20 dpc in the SS group while three of these also were different to controls in the RR group (Figure 2). As shown in the expression pattern of Figure 4 there were 45 miRNAs from the SS group that continued to decrease their expression at 20 dpc while 17 of the DE miRNAs increased their expression. The three miRNAs significantly different to controls in the RR group at 20 dpc (Figure 2) were all decreased and not different to the SS group. In summary, at 20 dpc the RR group changed toward the expression levels of controls while the SS group changed further away from controls (both decreases and increases) and showed differences that were significantly different to both controls as well as to the RR group.

### miRNAs Associated With IPN-QTL Genotype and Prolonged High Viral Loads

The comparisons of the relative expression of miRNAs between the two IPN-QTL genotype groups revealed no differentially expressed miRNAs between the SS and RR in the control group, at 1 dpc or at 7 dpc despite the observed difference in viral loads associated with the IPN-QTL genotype at 7 dpc (Figure 1). However, the comparison at 20 dpc revealed 38 miRNAs, from 18 miRNA families, that were significantly different expressed when comparing the SS group with high viral load and the RR group with low viral load. Ten of these mature miRNAs from five miRNA gene families were not observed in the time point comparisons of challenged fry vs. controls (RR or SS). These were: ssa-let-7a-1-3-3p, ssa-let-7b-3p, ssa-let-7d-c-1-3p, ssa-miR-7a-2-6-3p, ssa-miR-18b-3p, ssa-miR-29b-1-5p, ssa-miR-29b-2-5p, ssa-miR-29b-3-5p ssa-miR-29e-5p, and ssa-miR-8159-5p. The 28 remaining DE miRNAs were among the 72 DE miRNAs also showing significant differences when the challenged fry from each time point were compared to controls (Figure 2). The complete results on the 38 miRNAs showing different expression in comparison of RR and SS groups at 20 dpc and associated with prolonged differences in viral load, hereafter termed as high viral load responding miRNAs (HVL-DE miRNAs) are given in Table S3. This table shows the comparisons of the SS group against controls, the RR group against controls and the SS group against the RR group.

The miRNA expression differences between the SS group and the RR group leading to 38 HVL-DE miRNAs at 20 dpc were in most cases due to the SS group displaying changes that were more pronounced when compared to the controls than the RR group. While the low viral load RR group showed small changes compared to controls (mostly non-significant), the magnitude of change (increase or decrease) in the SS group with high viral loads was larger. In addition, some miRNAs discovered in the SS vs. RR comparison changed expression in different directions when compared to the controls. While these miRNAs were not significantly changed when either SS or RR groups were compared to controls, they were significant when SS and RR were compared to each other.

**Figure 5:**
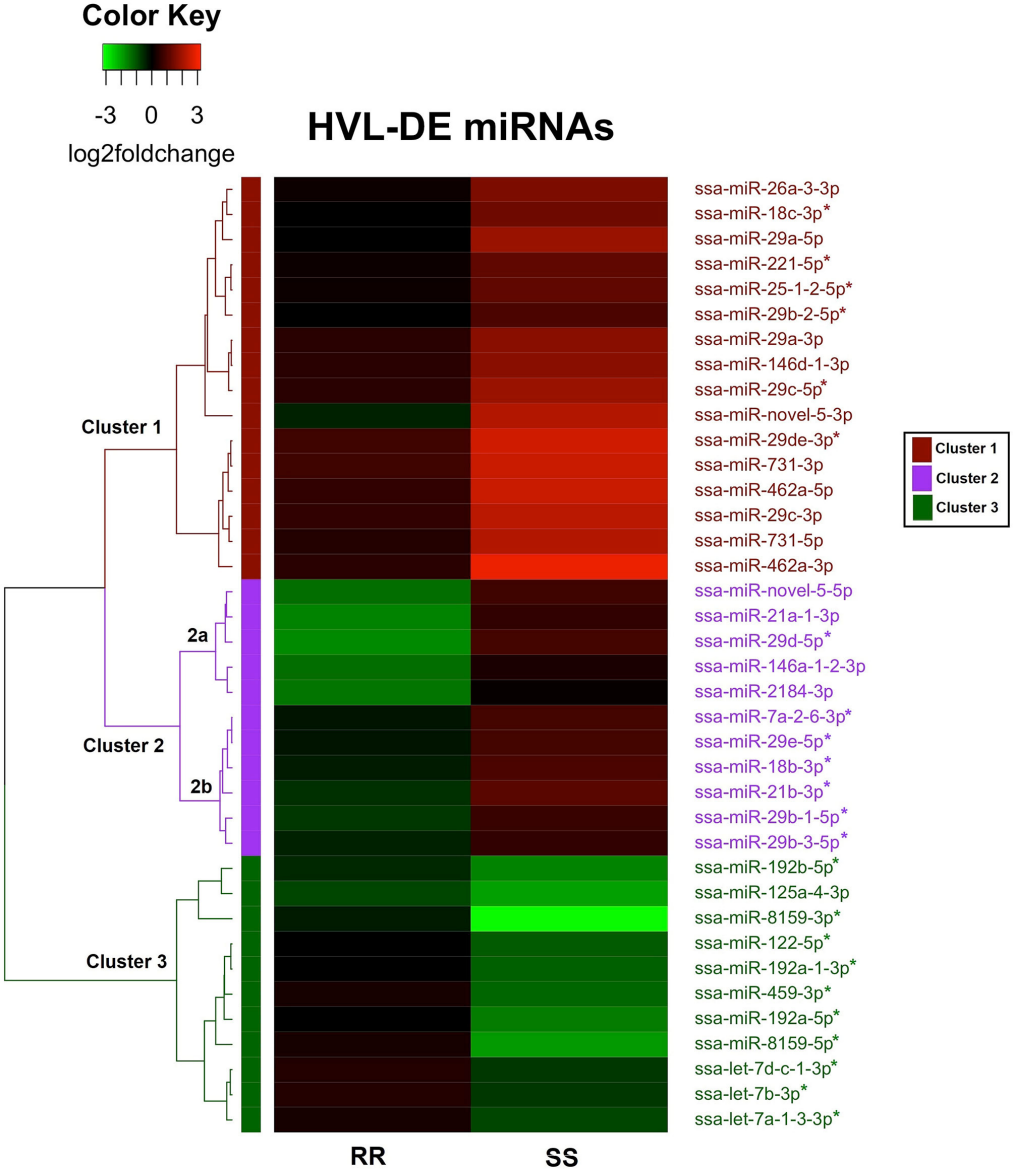
Heat map and hierarchical clustering of the 38 HVL-DE miRNAs. Each row represents a miRNA, and the columns represents the expression changes (log2foldchanges) in RR and SS groups at 20 dpc compared to controls. The dendrogram on the left shows the three major clusters of the HVL-DE miRNAs. The direction of the miRNA expression changes (log2foldchanges) are illustrated on the color key. Black color represents the expression in controls (baseline expression), red color indicates increased expression and green color indicates decreased expression. The color bars on the right indicate the three major clusters in which the HVL-DE miRNAs with similar expression profiles were grouped.

The changes in the 38 HVL-DE miRNAs were further examined by unsupervised hierarchical clustering analysis of the miRNAs differently expressed between RR and SS at 20 dpc. This analysis showed that there were three major clusters as shown in the heatmap in Figure 5. The miRNAs in Cluster 1 were characterized by larger increases in the SS group with higher viral loads, while the changes in the low viral load RR group were not significantly different to controls. There were sixteen miRNAs from nine families in this cluster including the ssa-miR-29, 462, and 731 families.

The second cluster included 11 miRNAs with expression changing in different direction in RR and SS groups compared to controls. As these changes were small when compared to healthy controls, most of these were not significantly different in the time point analysis at 20 dpc (Figure 2). There were five miRNAs in this cluster with pronounced decreased expression in RR and non-significant increased expression in SS, compared to the controls (ssa-miR-novel-5-5p, ssa-miR-21a-1-3p, ssa-miR-29d-5p, ssa-miR-146a-1-2-3p, and ssa-miR-2184-3p). These five miRNAs grouped together in a sub-cluster within cluster 2 (shown as 2a, Figure 5). The remaining six miRNAs in cluster 2 (shown as sub-cluster 2b, Figure 5) were slightly downregulated in the RR group and slightly upregulated in the SS group.

The HVL-DE miRNAs included in cluster 3 were those characterized by large decreases in the SS group, while they changed toward baseline expression that was not significantly different to controls in the RR group. There were eleven miRNAs from six familes in this cluster including the ssa-miR-192, miR-459, and miR-8159 families.

In summary, the analysis of QTL-groups at 20 dpc revealed 38 miRNAs differentially expressed between the RR and the SS groups. Compared to RR (and controls) there were 16 miRNAs with large increases in the SS group (cluster 1), while there were 11 miRNAs with large decreases (cluster 3). The remaining eleven miRNAs showed changes in different direction in RR and SS groups when compared to the baseline level of the controls. These miRNAs that were associated with QTL genotype are the ones that could contribute to difference in resistance/susceptibility to IPN.

### Differentially Expressed miRNAs May Regulate Immune Response Pathways

#### *In silico* Predictions of Target Genes

A means to better understand the functional significance of a particular set of miRNAs showing differential expression is to predict their target gene(s). Using *in silico* target gene analysis we predicted the target genes of all the differentially expressed miRNAs identified. A total of 2,434 different target transcripts were predicted. A complete list of all predicted targets along with the targeting DE miRNA and/or HVL-DE miRNA is given in Table S4.

The GO annotations retrieved from the Uniprot database (43) were used to limit our putative target gene set to include only those transcripts with functional annotation associated with immune system processes. There were 180 such putative target genes. They were either Atlantic salmon virus responsive genes (VRG) (45), or genes associated with immune responses [e.g., interferon regulatory factors (IRFs), C-C motif chemokines), inflammation and/or apoptotic processes (e.g., tumor necrosis factors (TNFs)]. All these genes are hereafter referred to as “immune genes” to simplify the text flow. Table S5 shows these immune genes along with the targeting DE miRNAs and/or HVL-DE miRNAs (differently expressed between RR and SS) shown in separate rows. Hundred and seventy of these genes were targeted by DE miRNAs while 148 genes were targeted by the 38 HVL-DE miRNAs.

A single miRNA may target several transcripts, and there was a wide distribution in number of targets for the individual miRNAs. Four of the miRNAs, ssa-miR-30d-2-3p, ssa-miR-21a-2-3p, ssa-miR-30c-d-1-3p, and ssa-miR-183-5p, showed the largest number of target matches (19, 17, 16, and 16 genes, respectively). Seventeen other miRNAs (ssa-miR-10b-5p, ssa-miR-10d-5p, ssa-miR-29a-3p, ssa-miR-29a-5p, ssa-miR-29b-3p, ssa-miR-29c-3p, ssa-miR-125b-3-3p, ssa-miR-129-5p, ssa-miR-143-3p, ssa-miR-146d-1-3p, ssa-miR-187-5p, ssa-miR-462a-3p, ssa-miR-727b-3p, ssa-miR-731-5p, ssa-miR-8162-5p, ssa-miR-novel-2-3p, and ssa-miR-novel-5-3p) also targeted from ten to fifteen of the immune genes. The remaining DE miRNAs could target from one up to nine of the immune genes. In the HVL-DE miRNA group the ssa-miR-8159-5p and ssa-miR-21b-3p targeted the largest number of genes (19 and 18 genes, respectively). Other miRNAs with a larger number of targets in the HVL-DE miRNA group were ssa-miR-731-5p (targeting 15 immune genes), and ten members of the miR-29 family targeting from 6 to 14 genes.

#### Immune Response Pathways Associated With the Targeted Immune Genes

Together the DE miRNAs and the smaller group of 38 HVL-DE miRNAs were predicted to target several key genes involved in the induction of Type I interferons (IFN-I), as well as pro-inflammatory cytokines, which play major roles in the defense response against viral infection in vertebrates (46, 47). Enrichment analysis with the 180 putative target genes as input were used to rank the gene pathways associated with all DE miRNAs while an additional enrichment analysis was carried out with the 148 predicted targets of the HVL-DE miRNAs (differently expressed between RR and SS groups). The results from these analysis' are given in Table S6. Seven KEGG pathways were those most enriched in target genes, each with combined scores above 120 and p-adjusted values (Q score) <1.0^−5^ (Table S6). The enrichment analysis with target genes predicted from the HVL-DE miRNAs also showed significant hits to the same seven pathways. The seven pathways were: the toll-like receptor signaling, the nucleotide-binding oligomerization domain (NOD)-like receptor signaling, the cytokine-cytokine receptor interaction, the apoptosis and the necroptosis pathways, the retinoic acid-inducible gene I (RIG-I)-like receptor signaling and the C-type lectin receptor (CLR) signaling. These pathways and the predicted target genes are given in Figures S1–S3. Table 1 shows the number of target genes predicted by all DE miRNAs or only by the HVL-DE miRNAs for each of the gene pathways. Table 2 shows the predicted genes in each pathway while their full names and GenBank accession numbers are given in Table S5. As shown in Table 2, several of the genes participate in more than one of these pathways. We also note that different paralogues e.g., the three Tumor necrosis factor alpha paralogues are targeted by different DE miRNAs (see Table S5).

**Table 1:** Gene pathways and number of targets predicted by all DE miRNAs vs. HVL-DE miRNAs.

**KEGG pathway**	**No of predicted target by all DE miRNAs**	**No of predicted targets by HVL-DE miRNAs**
sasa04620 Toll-like receptor signaling	20	15
sasa04621 NOD-like receptor signaling	21	18
sasa04060 Cytokine-cytokine receptor Inter.	24	18
sasa04210 Apoptosis	15	9
sasa04622 RIG-I-like receptor signaling	12	11
sasa04625 C-type lectin receptor signaling	13	10
sasa04217 Necroptosis	13	11

**Table 2:** Predicted target genes in the top seven enriched gene pathways.

**KEGG pathway^**a**^**	**Genes names^**b**^**
sasa04620: Toll-like receptor signaling	CATK; **CXL10**; **IKKA**; IRAK1; **IRF3**; **IRF5**; **IRF7**; **M3K8**; **MAPK**; MK03; **MK13**; **MP2K2**; **MP2K4**; **MYD88**; STAT1; **TBK1**; **TL/LRR**; **TNF-A**; **TNF-ALPHA-1**; TNF-ALPHA-2
sasa04621: NOD-like receptor signaling pathway	**BRCC3**; **CATB**; **DNM1L**; HS90A; **HSP90AB1**; **IKKA**; **IRF3**; **IRF7**; **IRF9**; **MAPK**; MK03; **MK13**; **MYD88**; **RIPK4**; STAT1; STATT2; **TBK1**; **TNF-A**; **TNF-ALPHA-1**; TNF-ALPHA-2; **TXNIP**
sasa04060: Cytokine-cytokine receptor interaction	**CCL19**; **CCR6**; **CCR9**; CD4; CD40L; **CXCR3**; CXCR4; **CXL10**; **CXL14**; **I10R2**; I12R2; **I13R2**; **IL-1RL**; **IL2RB**; **IL31R**; **IL4RA**; **TNF-A**; **TNF-ALPHA-1**; TNF-ALPHA-2; **TNF13**; TNR14; **TNR1A**; **TNR6**; **TR11B**
sasa04210: Apoptosis	CASP3; **CATB**; **CATF**; CATK; CATL1; CATSNE; **IKKA**; MCL-1; MK03; **MP2K2**; **TNF-A**; **TNF-ALPHA-1**; TNF-ALPHA-2; **TNR1A**; **TNR6**
sasa04622: RIG-I-like receptor signaling pathway	**CXL10**; **DHX58**; **IKKA**; **IRF3**; **IRF7**; **MAPK**; **MK13**; **SIKE**; **TBK1**; **TNF-A**; **TNF-ALPHA-1**; TNF-ALPHA-2
sasa04625: C-type lectin receptor signaling pathway	L1; C209A; IKKA; **IRF9**; **MAPK**; MK03; **MK13**; **NFKB2**; STAT1; STAET2; **TNF-A**; **TNF-ALPHA-1**; TNF-ALPHA-2
sasa04217: Necroptosis	**DNM1L**; HS90A; **HSP90AB1**; **IRF9**; **RIPK4**; STAT1; STAET2; **STAT4**; **TNF-A**; **TNF-ALPHA-1**; TNF-ALPHA-2; **TNR1A**; **TNR6**

a The identification number and the name of the KEGG pathways to which the genes have been mapped (44).

b *Target genes names are from the Uniprot database (43), all in bold font are targets of HVL-DE miRNAs. The GenBank accession numbers are given in Table S5 along with full gene names*.

The cytokine-cytokine receptor interaction pathway had 24 genes that could be targeted by the DE miRNAs while 18 of these genes were predicted as targets for the miRNAs differently expressed between RR and SS (HVL-DE miRNAs) (see also Tables 1, 2, and Table S5). Among these target genes were different chemokine receptors and their ligands that are important regulators of the immune response (48). The chemokine C-X-C motif chemokine 10 (CXL10, also known as IP-10) has been shown to be induced by IFN gamma, poly I:C and viral infections in Atlantic salmon (49–51). This chemokine participating in three pathways (Table 1) was predicted as target for ssa-miR-21b-3p, one of the 38 HVL-DE miRNAs, and the Atlantic salmon specific miRNA, ssa-miR-novel-13-5p (Table S5). Moreover, 20 genes that participate in the Toll-like receptor signaling and 21 genes that participate in the NOD-like receptor signaling pathways were putative targets for the DE miRNAs while this was 15 and 18 genes, respectively from the HVL-DE miRNAs (Tables 1, 2, and Table S5). Among the target genes in the NOD-like receptor signaling pathway was myeloid differentiation primary response protein 88 (MYD88) that was targeted by two HVL-DE miRNAs (ssa-miR-29a-5p and ssa-miR-29e-5p). The Atlantic Salmon MYD88 has been shown to interact with Interferon Regulatory Factor 3 (IRF3) and 7 (IRF7), to modulate IRF-dependent antiviral IFN response (52) (see below).

The RIG-I-like receptor signaling pathway had twelve genes that could be targeted by the DE miRNAs and except one of the TNF-A paralogs they were also predicted as targets for the HVL-DE miRNAs (Tables 1, 2, and Table S5). The suppressor of IKK-epsilon (SIKE), a negative regulator of the RIG-I-pathway (53, 54), was predicted as a target for 15 miRNAs from 11 miRNA families (ssa-let-7a-1-3-3p, ssa-miR-10b-5p, ssa-miR-10d-5p, ssa-miR-21a-2-3p, ssa-miR-21b-3p, ssa-miR-26a-3-3p, ssa-miR-29e-5p, ssa-miR-143-3p, ssa-miR-146a-1-2-3p, ssa-miR-146a-3-3p, ssa-miR-146b-3p, ssa-miR-183-5p, ssa-miR-8159-5p, ssa-miR-novel-2-3p, and ssa-miR-novel-5-3p). Another interesting target was the IRF3 that interacts with MYD88. IRF3 is the main regulator of salmon IFN production (47, 55). This key gene, participating in three pathways (Table 1), was predicted as target for fourteen miRNAs from seven families (ssa-miR-10 family, ssa-miR-18b-3p, ssa-miR-21 family, ssa-miR-29 family, ssa-miR-30 family, ssa-miR-8159-5p and ssa-miR-novel-5-3p). The same members of the ssa-miR-30 family (ssa-miR-30c-d-1-3p and ssa-miR-30d-2-3p) could also target IRF7, another MYD88 interacting IRF in this pathway. In addition, three other miRNAs (ssa-miR-192a-5p, ssa-miR-459-3p and ssa-miR-731-5p) also targeted IRF7.

Apoptosis and necroptosis (programmed necrotic cell death) are important host response mechanism to counteract invading pathogens, including viruses (56–58). Twenty-three different genes that participate in apoptosis and/or necroptosis pathways could be targeted by the DE miRNAs while 16 of these were also targets of the HVL-DE miRNAs (Tables 1, 2 and Table S5). Among the target genes involved in both pathways were two death receptors of the tumor necrosis factor receptor (TNFR) superfamily, Tumor necrosis factor receptor superfamily member 6 (TNR6) and Tumor necrosis factor receptor superfamily member 1A (TNR1A) (56, 59), that were targeted by seven (ssa-miR-10d-5p, ssa-miR-100a-2-3p, ssa-miR-187-5p, ssa-miR-192a-5p, ssa-miR-726-3p, ssa-miR-727b-3p, and ssa-miR-novel-13-5p) and five miRNAs (ssa-miR-18b-3p, ssa-miR-30c-d-1-3p, ssa-miR-30d-2-3p, ssa-miR-183-5p, and ssa-miR-novel-5-5p), respectively. The large number of targets predicted from these two pathways suggest that the DE miRNAs, including some HVL-DE miRNAs, are involved in regulation of key genes that control programmed cell death (Apoptosis and Necroptosis pathways, Figure S3).

#### Immune Genes Targeted by HVL-DE miRNAs With Changed Expression at 20 dpc

The relationship between a differentially expressed miRNA and its predicted target may be explored by additional analysis of target gene expression. The gene expression in the materials investigated in this study was examined in parallel by microarray analysis (unpublished work by Dr. Diego Robledo and colleagues). Twenty-five of the 180 immune genes predicted as targets of the HVL_DE miRNAs were revealed as differentially expressed in the SS group vs. the RR group at 20 dpc by microarray analysis. These genes and the HVL-DE miRNAs (different expression between RR and SS group at 20 dpc) that were predicted to target the genes are shown in Table 3. Results from microarray analysis revealed that all 25 genes showed higher expression in the SS group vs. the RR group. In most cases the HVL-DE miRNAs showed similar change as the target genes (i.e., higher in the SS group vs. the RR group at 20 dpc). Although it is expected that a miRNA regulating a target gene would show opposite change in expression to its target, the changes in similar direction could be explained by models where miRNAs fine-tune target gene expression [see discussion and Andreassen and Høyheim (13)].

**Table 3:** Immune genes differentially expressed between the RR and SS groups that were predicted as targets of the HVL-DE miRNAs at 20 dpc.

**Target gene^**a**^**	**Immune function^**b**^**	**HVL-DE miRNAs, SS, vs. RR^**c**^**
Toll-like leucine-rich repeat protein (TL/LRR)	Activator, inflammatory responses to pathogens, Innate immunity	↑ miR-731-5p
Signal transducer and activator of transcription 3 (STAT3)	Activator, acute-phase response, host-virus interaction, regulation of type I interferon signaling pathway, viral process	↓ let-7d-c-1-3p ↑ ssa-miR-18b-3p
Signal transducer and activator of transcription 2 (STAT2)	Activator, antiviral defense, host-virus interaction, cytokine-mediated signaling pathway, regulation of type I interferon signaling pathway, viral process	↓ let-7d-c-1-3p, ↑ miR-18b-3p
Tumor necrosis factor alpha-1 (TNF-alpha-1)	Activator, inflammatory response, I-kappaB kinase/NF-kappaB signaling, extrinsic apoptotic signaling pathway via death domain receptors, humoral immune response	↑ miR-novel-5-3p
E3 ubiquitin-protein ligase TRIM39-like (LOC106608979)	Activator, positive regulation of apoptotic signaling pathway	↑ miR-7a-2-6-3p
Interferon regulatory factor 3 (IRF3)	Activator, transcription factor activating innate immunity pathway, antiviral defense against DNA and RNA viruses, host-virus interaction	↓ miR-8159-5p ↑ miR-18b-3p ↑ miR-21b-3p ↓ ↑ miR-29a-3p ↑ miR-29b-3-5p ↑ miR-29de-3p ↑ miR-29c-3p ↑ miR-novel-5-3p
Interferon regulatory factor 7 (IRF7)	Activator, transcription factor activator that promote inflammation, antiviral defense against DNA and RNA viruses, critical for the late than for the early phase of the IFN gene induction.	↓ miR-192a-5p ↓ miR-459-3p ↑ miR-731-5p
C-C motif chemokine 19 (CCL19)	Cytokine, virus responsive gene, immune response, Inflammatory response, innate immunity pathway	↑ miR-26a-3-3p ↑ miR-731-3p
Thioredoxin-interacting protein (TXNIP)	Transcriptional repressor, required for the maturation of natural killer cells, positive regulation of apoptotic process	↓ let-7a-1-3-3p ↓ miR-8159-3p
Angiopoietin-related protein 4 precursor (ANGL4)	Inhibitor, negative regulation of apoptotic process	↓ miR-192a-1-3p ↓ miR-192b-5p ↑ miR-29a-5p ↑ miR-29e-5p ↑ miR-7a-2-6-3p
C-X-C motif chemokine 10 precursor (CXL10)	Pro-inflammatory cytokine, involved in chemokine-mediated signaling pathway, immune response	↑ miR-21b-3p ↓
C-X-C chemokine receptor type 3 (CXCR3)	Receptor for C-X-R chemokines, regulates biological processes such as immune response, inflammatory, response, and apoptotic process	↓ miR-8159-5p ↑ miR-731-5p (2x) ↑ miR-7a-2-6-3p
Interleukin-4 receptor alpha chain precursor (IL4RA)	Receptor, cytokine-mediated signaling pathway, immune response, production of molecular mediators involved in inflammatory response	↑ miR-novel-5-3p
Interleukin-10 receptor beta chain precursor (I10R2)	Receptor, virus responsive gene, cytokine-mediated signaling pathway, antiviral defense response, immune response, inflammatory response	↑ miR-18b-3p ↑ miR-221-5p ↑ miR-29b-2-5p ↑ miR-29c-5p (2x)
Probable ATP-dependent RNA helicase DHX58 (DHX58)	Virus responsive gene, host-virus interactions, innate immune response to RNA viruses and some DNA viruses such as poxviruses, negative regulator of DDX58/RIG-I and IFIH1/MDA5 mediated antiviral signaling	↑ miR-731-5p
Interferon-induced protein with tetratricopeptide repeats 5-like (IFIT5)	Virus responsive gene, defense response to virus, innate immune response, negative regulation of viral genome replication, positive regulation of I-kappaB kinase/NF-kappaB signaling	miR-2184-3p ↓
XIAP-associated factor 1 (XAF1)	Virus responsive gene, pro-apoptotic gene, regulation of apoptotic process	↓ miR-122-5p ↑ miR-146d-1-3p ↑ miR-29b-3-5p
Macrosialin precursor (CD68)	Virus responsive gene	↓ let-7b-3p ↑ miR-731-5p ↑ miR-462a-3p (5x)
Interferon-induced transmembrane protein 5 (IFM5)	Virus responsive gene	↑ miR-21b-3p ↓
Receptor-transporting protein 3 (RTP3)	Virus responsive gene	↑ miR-29a-3p, ↑ miR-29de-3p, ↑ miR-29c-3p
IFN-inducible protein Gig2-like-1	Virus responsive gene	↑ miR-462a-3p
Interferon-induced protein 44 (IFI44)	Virus responsive gene, antiviral defense, immune response	↑ miR-21b-3p ↓
Barrier-to-autointegration factor (BAF)	Virus responsive gene, host-virus interaction, known be exploited by retroviruses to facilitate integration of retroviruses in host genomes	↑ miR-21b-3p ↓
VHSV-induced protein-like (LOC100194553)	Virus responsive gene, immune response	↑ miR-221-5p, ↑ miR-29b-1-5p ↓ ↑ miR-29c-5p miR-29d-5p ↓
VHSV-inducible protein (VHSV)	Virus responsive gene, upregulated in VHSV	↓ let-7d-c-1-3p, miR-29d-5p ↓

a Target genes names as annotated in the Uniprot database (43).

b The description of immune functions are based on GO terms in the Uniprot database and Krasnov et al. (45).

c *HVL-DE miRNAs with target site matches in the 3'UTRs of the target genes. For space saving reasons the ‘ssa'prefix was removed. Upward arrows (↑) illustrate higher expression, and downward arrows (↓) indicate lower expression in the SS group vs. the RR group at 20 dpc. In cases where the HVL-DE miRNAs showed large changes in different directions, the arrow prior to miRNA name denotes change in SS, and arrow behind miRNA denotes change in RR (Figure 5). If a miRNA has multiple matches to a target this is indicated in the following brackets*.

Eleven of the 25 genes were targeted by only one miRNA. It is, however, shown that a target gene may be regulated by several miRNAs (60, 61). If one gene is targeted by several miRNAs that change their expression in a similar manner, this would add evidence that it is a true target gene. Five such target genes were revealed. These were the C-C motif chemokine 19 (CCL19), the Thioredoxin-interacting protein (TXNIP), the Interleukin-10 receptor beta chain precursor (I10R2), the Receptor-transporting protein 3 (RTP3) and the VHSV-induced protein-like (LOC100194553) (Table 3). Other targets also showed matches to several miRNAs, but often with one miRNA changing expression in opposite direction vs. the others. This could reflect a falsely predicted target site or that the target gene regulation is more complex and may be fine-tuned by miRNAs changing in different directions.

## Discussion

IPNV is an important viral pathogen that has had a major impact on salmonid aquaculture. Analysis of the IPNV challenge materials investigated here allowed us to compare viral load and miRNA expression in family-matched fry with alternate genotypes [IPN-QTL susceptible (SS) or IPNV-QTL resistant (RR)]. Significant viral loads were detected in equal amounts in both IPN-QTL genotypes (RR and SS) at 1 dpc. The absence of an early difference (1 dpc) in viral loads between any of the individuals is in agreement with other studies (21, 27).

The susceptible genotypes showed much higher average viral load than resistant genotypes at 7 and 20 dpc. This is also in agreement with the viral load measurements in Robledo et al. (21). Some controls showed positive, but very low amounts of IPNV. They were still included as we assumed this was contamination at challenge initiation, and the higher number of controls would lead to a better estimate of variation. The controls were sampled prior to challenge initiation and challenged samples from 1, 7, and 20 dpc were compared to these. The 38 HVL-DE miRNAs differentially expressed in the SS group were, however, not significantly different expressed when comparing the low-viral RR-group with the controls. This indicates that the miRNA expression changes reflect differences in response to IPNV rather than being a time related bias.

Previous studies have indicated that host-cell miRNAs play a key role in fine-tuning Atlantic salmon immune response to viral infection (12, 13). In the present study, we identified miRNAs that showed expression changes in response to IPNV at different time points compared to controls, but not necessary different between RR and SS groups (DE miRNAs). The other group of miRNAs that could affect the resistance/susceptibility where those that showed differences between the SS group with high viral loads and the RR groups with low viral load (HVL-DE miRNAs). These were only detected at 20 dpc. Finally, we explored the role of the DE miRNAs in the immune response by *in silico* prediction of their immune-relevant target genes. The analysis of miRNA expression in the time point analysis identified 72 Atlantic salmon miRNAs that showed dynamic expression changes indicating a temporal regulation of the miRNA responses following IPNV challenge (DE miRNAs, Figure 2). Similar dynamic changes involving many of the same miRNAs were observed in a challenge study with Salmonid Alpha virus (SAV) (12). The 72 DE miRNAs could, in general, be divided into two groups; those with downregulated expression in challenged groups between 7 and 20 dpc (Figures 3, 4), and those that showed upregulated expression at 20 dpc (Figures 3, 4). The fact that many of the same miRNAs responded to IPNV challenge in a similar manner as to SAV challenge (12) indicates that they are part of the general immune response to viral infection rather than a particular response to IPNV (or SAV). Moreover, some conserved miRNAs (e.g. miRNAs from families 21, 146, 181, 462, and 731) have shown similar responses to viral challenge in many different teleosts (13, 62). This further indicates that they contribute to the regulation of common immune and anti-viral response genes. However, the particular response and magnitude of change in different teleost fish could depend, and be manipulated by, the pathogen (13, 62, 63).

There was a clear difference in response to infection between the high viral load SS group and the low viral load RR group. However, the differences in miRNA expression between the SS group and the RR group (HVL-DE miRNAs) were only detected at the latest time point (i.e., 20 dpc). Thus, this appears as a late response to the more severe viral infection in the HVL individuals (SS group showing much higher viral loads from 7 dpc). The 38 HVL-DE miRNAs could be clustered into three groups: miRNAs responding to viral infection with more pronounced changes (either increases or decreases) in the SS group vs. controls (Cluster 1 and 3, Figure 5), or miRNAs with expression changes in different directions in the HVL-SS and the LVL-RR groups compared to controls (Cluster 2, Figure 5). This last group is interesting, as these miRNAs, including the miR-21, miR-146 and miR-2184 family members, would contribute opposite regulation of their target genes compared to the expression level of the non-infected samples in the later phase of the inflammatory response. Since the HVL-DE miRNAs were the ones differently expressed between the RR and SS group it is possible that they contribute to differences in resistance/susceptibility, but as in any study revealing association, the association alone is not enough to verify causation.

**Figure 6:**
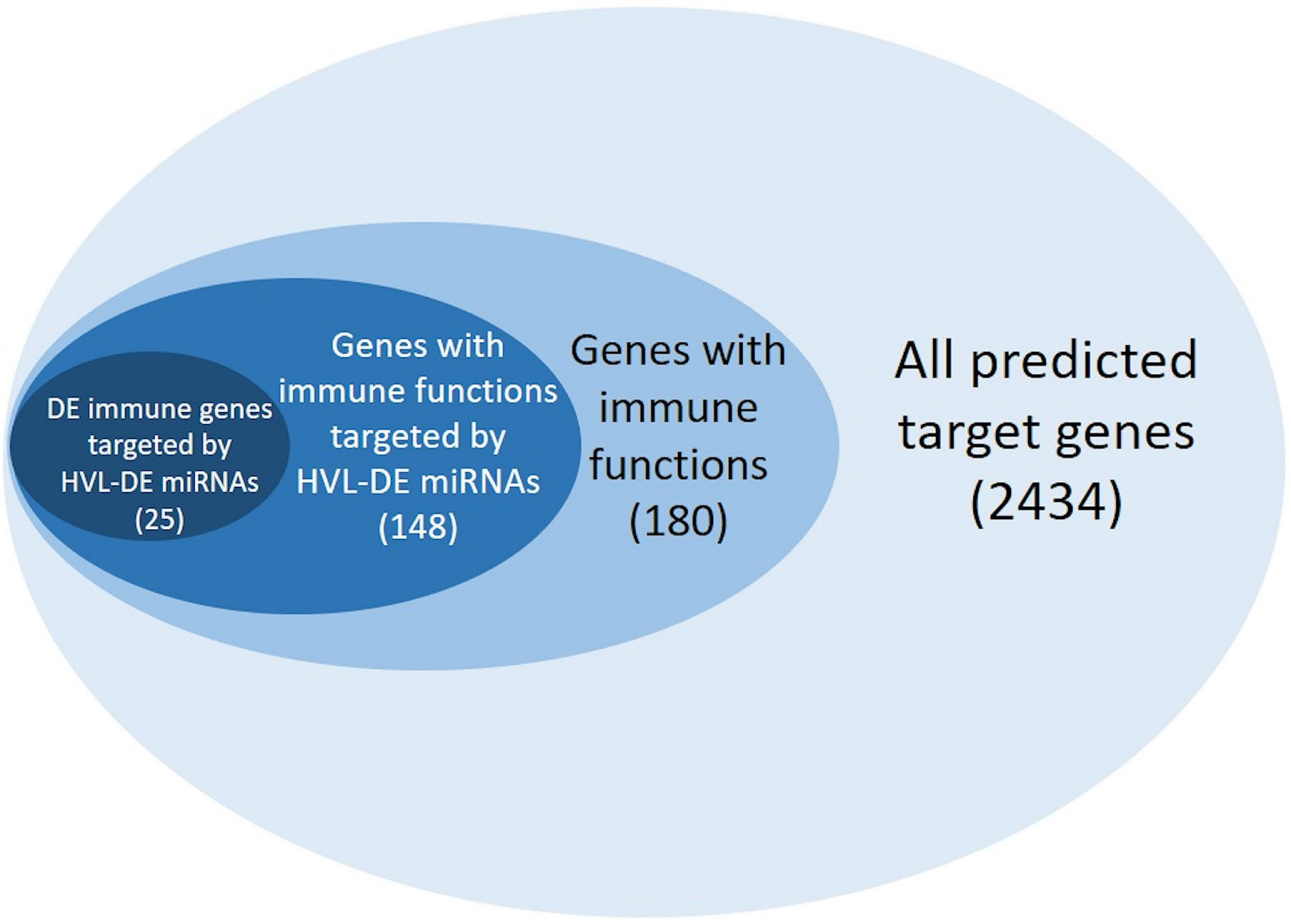
Number of miRNA targets identified by our stepwise approach (*n* = 2,434). The targets with GO terms associated with immune functions are shown in light blue (*n* = 180), while the immune genes showing different expression in comparison of the RR groups and SS groups (*n* = 148) are shown in blue, and finally the immune genes differently expressed at 20 dpc (microarray analysis) and predicted as target for HVL-DE miRNAs are shown in dark blue (*n* = 25).

Analysis of potential target mRNAs is essential for revealing the biological processes and to identify the gene pathways that are regulated by miRNAs. The target genes may be predicted by *in silico* analysis where the putative target sites in the 3'UTRs are identified. Such predictions commonly result in many false positives when the complete transcriptome is used as input (13). Here, we applied a stepwise approach to single out the most likely targets (Figure 6). First the transcriptome was used as input, then the target genes with immune related functions (immune genes) were singled out, and finally, those immune genes with significant expression differences between the SS group and the RR group at 20 dpc were identified. Assuming that the miRNAs do contribute to fine-tuning the immune response, the 25 immune genes changing their expression at the same stage in the same materials would be the most likely targets (Table 3). Studies have shown that several genes involved in type I IFN and antiviral immunity pathways participate in host response to IPNV (21, 26–29, 47), and several of the DE and HVL-DE miRNAs were predicted to target genes that regulate antiviral immunity pathways, such as those leading to Type I IFN response (e.g., IRFs and STATs) (Table 2). Interestingly, many of the genes participating in these pathways were also among the 25 genes differentially expressed between the SS and RR groups (Table 3). The functional annotation and enrichment analysis illustrated that the miRNAs responding to IPNV could contribute to the regulation of most of the important signaling pathways activated upon viral infection by targeting multiple genes as illustrated in the seven KEGG pathways (Figures S1–S3).

Interpretation of the function of each individual miRNA is complicated by the possibility that some targets are falsely predicted. It is also difficult to interpret because their expression changes from one time point to another post challenge, which leads to different effects on their target gene. In the proposed model for miRNA function in teleost fish immune homeostasis (13) we suggested that the dynamic expression of miRNAs changes according to what would benefit the host anti-viral defense during the infection. In this model, the miRNA and its target transcript do not necessarily show inverse proportional expression. In the late stage of viral infection, both a miRNA contributing to fine-tuning the expression of the inflammatory activators as well as the activators themselves could show increased expression. This could help fine-tune the elevated expression of the activator at a level where the inflammatory processes protect against virus but are not increased to levels where it is harmful to the host. A model with such multiple functions for several miRNAs is supported by findings in studies of innate immunity response and autoimmune diseases in higher vertebrates (64–66). Finally, the interpretation of miRNA function is complicated because the observed miRNA responses are from challenge studies. The pathogens cause disease as they have the ability to escape the host defense and sometimes manipulate the host response to benefit their own propagation (63, 67). Thus, what one observes may not be the miRNA expression change that serves the host, but in some cases, rather a pathogen-manipulated expression change that benefits pathogen propagation.

Nevertheless, several of the DE and HVL-DE miRNAs responded in accordance with the proposed dynamic model (13). They showed decreased expression at 7 dpc and their predicted target genes were part of signaling pathways that induce a host-protective response. Therefore, a decreased expression of miRNAs that target these genes would contribute to promote the immune response. Likewise, others that were upregulated at 20 dpc were predicted to target inflammatory activators, and an increased expression at 20 dpc could in these cases prevent further escalation of inflammatory processes.

Regulation of many predicted targets is not straightforward to interpret. For example, IRF3, a key gene in activation of immune responses showed increased expression in the SS (HVL) group vs. RR (LVL) group at 20 dpc (Robledo et al., unpublished) demonstrating that this gene is involved in response to IPNV infection. Fourteen miRNAs were predicted to target IRF3 (Table 4). The decreased expression of the six miRNAs [miR-10, miR-21, and miR-30 family members, two from each family (Table 4)] at 7 dpc could be a response to viral infection, and their decreases would contribute to activate the host antiviral responses by increasing the expression of IRF3 at this stage of infection. At 20 dpc eight miRNAs responded to the inflammatory level in the SS group by increasing their miRNA expression, while these changes did not occur in the RR group (Table 4). This would inhibit further increase of IRF3 expression in the SS group. These changes could help to contain and balance the level of inflammatory response, which may be harmful to the host over time. The decrease of ssa-miR-8159-5p only in the SS groups at 20 dpc does however, not fit the model. One explanation could be that IRF3 is a falsely predicted target for this miRNA. Nevertheless, IRF3 and the predicted miRNA interactions do illustrate that there might be complex and dynamic host-cell miRNA fine-tuning mechanisms acting during viral infections.

**Table 4:** Expression changes in the 14 miRNAs that were predicted to target IRF3.

**miRNAs^**a**^**	**1 dpc^**b**^**	**7 dpc^**b**^**	**20 dpc-SS^**c**^**	**20 dpc-RR^**c**^**
ssa-miR-10d-5p	0.7	−1.2	−1.4	−1.0
ssa-miR-10b-5p	0.7	−1.2	−1.4	−1.0
ssa-miR-30c-d-1-3p	0.7	−1.1	−1.2	−1.0
ssa-miR-30d-2-3p	0.7	−1.1	−1.2	−1.0
ssa-miR-21a-2-3p	0.4	−1.9	−1.4	−1.8
ssa-miR-21b-3p	0.4	−0.7	1.1	−0.6
ssa-miR-18b-3p	0.1	−0.1	1.0	−0.4
ssa-miR-novel-5-3p	0.5	−0.2	2.2	−0.5
ssa-miR-29b-3-5p	0.9	−0.2	0.6	−0.4
ssa-miR-29b-3p	−0.1	0.6	1.5	0.6
ssa-miR-29a-3p	−0.1	0.2	1.7	0.5
ssa-miR-29c-3p	0.2	0.4	2.3	0.6
ssa-miR-29de-3p	−0.4	0.5	2.5	0.7
ssa-miR-8159-5p	0.0	0.3	−1.9	0.2

a Mature Atlantic salmon miRNA names as annotated in Woldemariam et al. (33).

b log2 fold change from DESeq2 analysis of IPNV challenged samples vs. controls at 1 and 7 dpc, respectively.

c *log2 fold change from DESeq2 analysis of IPNV challenged SS groups vs. controls and RR groups vs. controls at 20 dpc, respectively*.

Another interesting result revealed was that paralogs could be regulated by different miRNAs. The salmon genome consists of a large number of closely related paralogs due to a salmonid-specific whole genome duplication that occurred ~80 million years ago (68, 69). Tumor necrosis factor (TNF) alpha is a pro-inflammatory cytokine that plays a key role in regulation of inflammation and immunity. Multiple paralogs of tumor necrosis factor alpha have been identified in teleost species, including Atlantic salmon (70, 71). Our predictions showed that TNF-ALPHA-2 was targeted by ssa-miR-222b-5p, while TNF-ALPHA-1- was targeted by ssa-miR-222b-5p and the species-specific miRNA ssa-miR-novel-5-3p. In contrast, TNFA was targeted by seven other miRNAs (ssa-miR-21a-2-3p, ssa-miR-21b-3p, ssa-miR-146d-1-3p, ssa-miR-183-5p, ssa-miR-192a-5p, ssa-miR-459-3p, and ssa-miR-8159-5p) (Table S5). This demonstrates that when genes undergo duplication, the extra gene copies are free to assume new functions including different regulatory responses (72). These results also demonstrate the importance of full-length transcript sequence information on paralogous genes to understand the miRNA mediated gene regulatory mechanisms.

In summary, although a number of immune genes were predicted as targets, often for several of the responding miRNAs, the target-miRNA interactions seem too complex to be fully understood from the expression analysis and the target predictions alone. Despite these limitations, the presented results showed that the DE miRNAs could together target genes that are part of several different gene pathways. Predicted targets were revealed in pathways that induce Type I interferons (IFN-I) (e.g., Toll-like receptor signaling, NOD-like receptor signaling and RIG-I receptor signaling) as well as apoptosis regulators. Most of these immune genes were also targeted by HVL-DE miRNAs which opens the possibility that some miRNAs contribute to differences in resistance/susceptibility at the later stages of infection. Our further studies will aim to investigate the predicted miRNA-target gene interactions from this study by functional approaches to uncover the role of the miRNAs in IPNV pathogenesis mechanisms.

## Data Availability Statement

The datasets generated for this study can be found in the NCBI Sequence Read Archive under project accession number PRJNA554632.

## Ethics Statement

The animal study was reviewed and approved by Centre for Environment, Fisheries and Aquaculture Science (CEFAS), Weymouth, Dorset.

## Author Contributions

RA and BH conceived and coordinated the study. RH and JB contributed all challenge samples. OA performed major part of the processing of the deep sequenced data and the DESeq2 analysis. HS performed RT-qPCR measurements of viral loads. RA and NW performed the data analyses and interpretation of the results. DR performed the microarray analysis. NW wrote the first draft of the manuscript under the supervision of RA. All authors contributed to manuscript revision, read and approved the final version.

## Conflict of Interest

The authors declare that the research was conducted in the absence of any commercial or financial relationships that could be construed as a potential conflict of interest.
