# Supplementary figures and images for: miRNAs Predicted to Regulate Host Anti-viral Gene Pathways in IPNV-Challenged Atlantic Salmon Fry Are Affected by Viral Load, and Associated With the Major IPN Resistance QTL Genotypes in Late Infection
Source: Front Immunol. 2020 Sep 11;11:2113. doi: 10.3389/fimmu.2020.02113 (PMC7516080; doi:10.3389/fimmu.2020.02113)

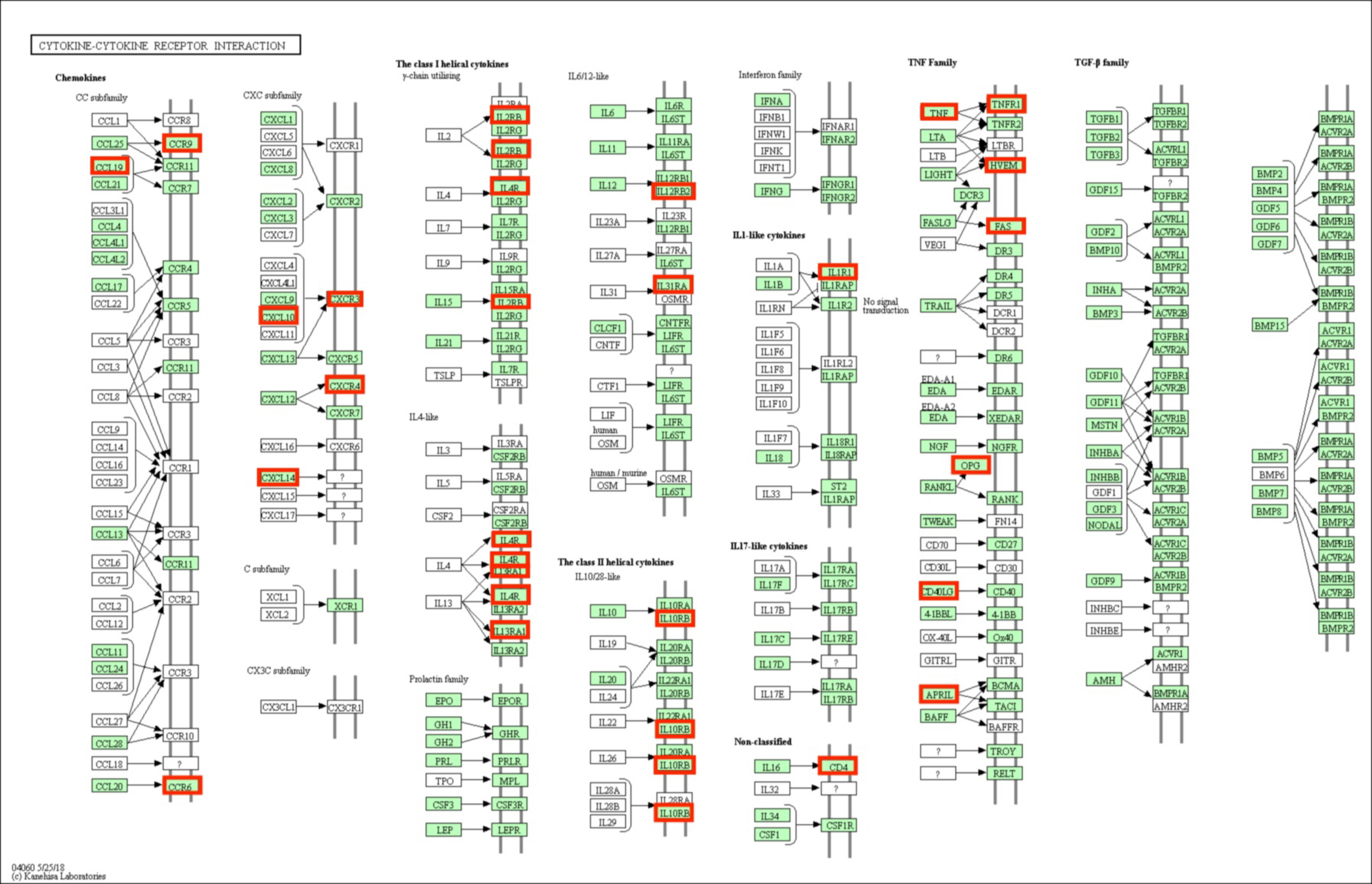

Supplement: Supplementary file 7 [file Image_1.JPEG]

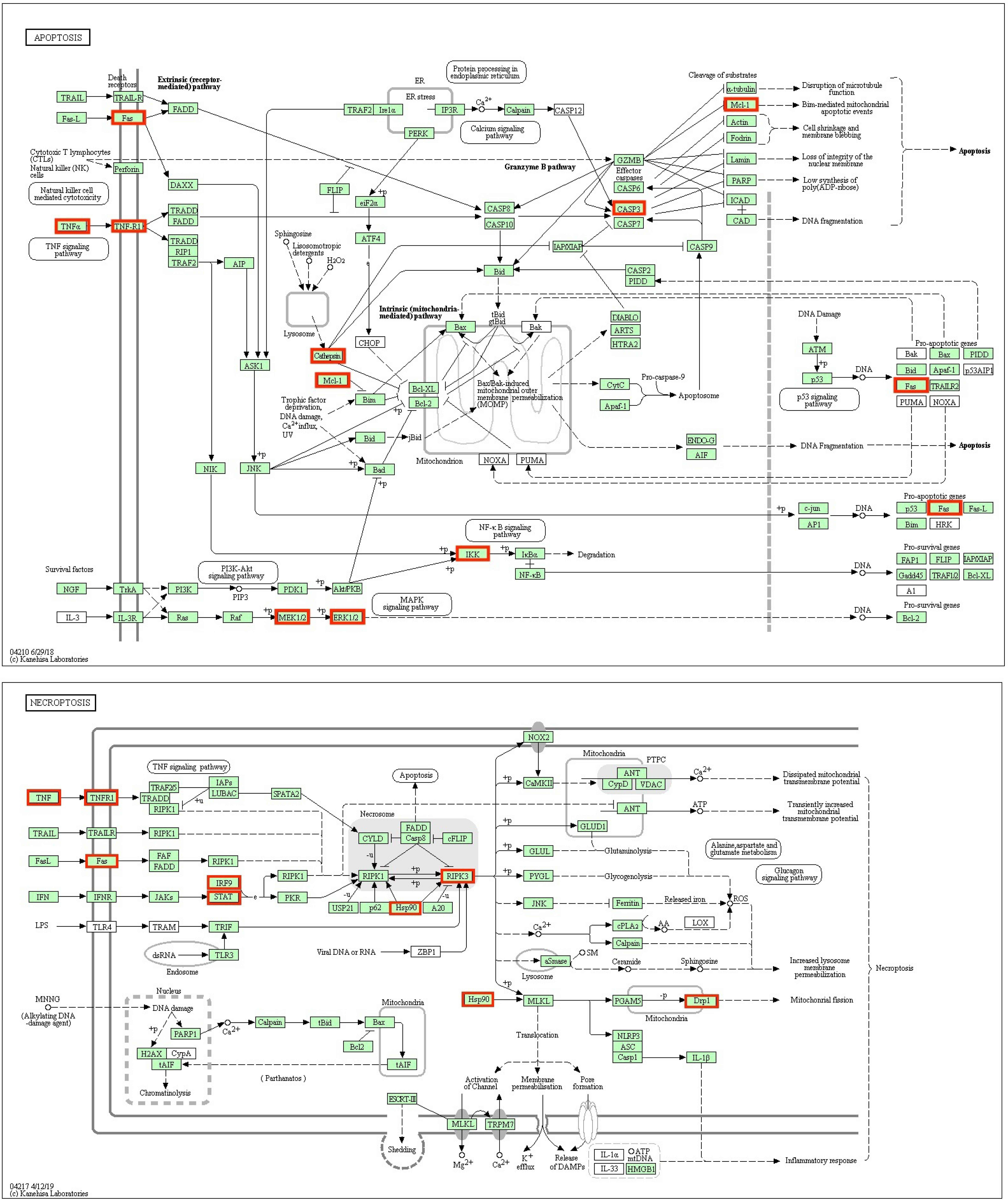

Supplement: Supplementary file 9 [file Image_3.JPEG]
